# Non-cytotoxic hydroxyl-functionalized exfoliated boron nitride nanoflakes impair the immunological function of insect haemocytes *in vivo*

**DOI:** 10.1038/s41598-019-50097-0

**Published:** 2019-10-01

**Authors:** Elżbieta Czarniewska, Lucyna Mrówczyńska, Magdalena Jędrzejczak-Silicka, Patryk Nowicki, Martyna Trukawka, Ewa Mijowska

**Affiliations:** 1 Department of Animal Physiology and Developmental Biology, Adam Mickiewicz University, Institute of Experimental Biology, Uniwersytetu Poznańskiego Str. 6, 61-614 Poznań, Poland; 2 Department of Cell Biology, Adam Mickiewicz University, Institute of Experimental Biology, Uniwersytetu Poznańskiego Str. 6, 61-614 Poznań, Poland; 3 Laboratory of Cytogenetics, West Pomeranian University of Technology, Klemensa Janickiego Str. 29, 71-270 Szczecin, Poland; 4 Nanomaterials Physicochemistry Department, West Pomeranian University of Technology, Piastów Avenue Str. 45, 70-311 Szczecin, Poland

**Keywords:** Immunological models, Focal adhesion, Small molecules

## Abstract

To induce the water solubility of hexagonal boron nitride (h-BN), we exfoliated and functionalized bulk h-BN with hydroxyl groups (h-BN-OH-n). Short-term studies showed that h-BN-OH-n induced low cytotoxicity in different models: insect haemocytes (*in vivo*), human erythrocytes and mouse fibroblasts (*in vitro*). We also demonstrated that Alexa Fluor 647-h-BN-OH-n administered topically to the insects passed through the cuticle barrier and was phagocytosed by haemocytes. Nanoflakes did not affect the haemocyte cell membrane and did not interfere with the phagocytosis of latex beads. Long-term immunoassays showed that h-BN-OH-n, despite not inducing haemocytotoxicity, impaired nodulation, the most important cellular immune response in insects. The haemocytes exposed to h-BN-OH-n and then to bacteria differed in morphology and adhesiveness from the haemocytes exposed only to bacteria and exhibited the same morphology and adhesiveness as the control haemocytes. The h-BN-OH-n-induced decrease in nodulation can therefore result from the reduced ability of haemocytes to recognize bacteria, migrate to them or form microaggregates around them, which can lead to dysfunction of the immune system during pathogen infection. Long-term *in vivo* studies with animal models are still necessary to unambiguously confirm that h-BN is biocompatible and useful for application as a platform for drug delivery or for bioimaging.

## Introduction

Boron nitride (BN), known as white graphene, is a structural analogue of graphene in which C atoms are replaced by alternating B and N atoms. Hexagonal boron nitride (h-BN) is a layered material with a graphite-like structure; it can form nanotubes with improved properties compared to carbon nanotubes^1^. h-BN exhibits outstanding electrical properties because it consists of approximately 50% N atoms, which differs from the comparison of graphene, such that it requires nitrogen doping for electrochemical applications. Therefore, h-BN is electrically insulating with a band gap of ~5–6 eV and has highly thermal properties (with a high thermal conductivity of 390 Wm^−1^K^−1^ in the basal plane), a high melting point, good resistance to corrosion, low density, and excellent mechanical properties (with a measured Young’s modulus of 1.22 ± 0.24 TPa) and high chemical stability^1–4^.

Despite the promising properties of h-BN and its potential utility due to, e.g., thermal and chemical stability, h-BN is not soluble in aqueous media, and its functionalization has been explored less and is more challenging compared with that of C-based materials^5,6^. The water solubility of h-BN can be enhanced using exfoliating methods^5^. It is known that h-BN preserves its hexagonal structure throughout the functionalization process and that the B–N bonds in the structure of h-BN have partial ionic characteristics^6,7^. The B atoms carry a partial positively charge (electron deficient centres), whereas the N atoms are negatively charged (electron rich centres). These characteristics of h-BN render the B site accessible for attack by nucleophilic groups. On the other hand, the N site reacts with electrophilic groups^6^. Thus, numerous recent studies have been conducted on h-BN functionalization with functional groups, e.g., hydroxyl (-OH), amino (-NH_2_), ether (-OR), amine (-NHR), acryl (-COR), alkyl (-R), halogen (-X) and heteroatoms (C and O)^6^. In other studies, boron nitride nanostructures were successfully non-covalently functionalized with synthetic polymers (e.g., poly(*p*-phenylene ethynylene) (PPE), a poly(*p*-phenylene) derivative ((-)PPP), poly(xylidine tetrahydrothiophene) (PXT), poly(sodium styrene sulfonate) (PSS), and poly(sodium vinyl sulfonate) (PVS), and poly(sodium acrylate) (PAA)) and with biomolecules (e.g., peptides, proteins, DNAs, RNAs, saccharides, lipids, and more complexes, such as glycodendrimers)^6,8,9^. These modifications can be efficiently used for dispersing and functionalizing processes to overcome the h-BN limitations during biological implementation^8,9^.

Data obtained in 2012 by the FDA indicate that BN is used in 483 cosmetics. The highest BN concentrations are found in eye shadows (up to 25%), powders (up to 16%) and lipsticks (up to 2%)^10^. The use of BN as a cosmetic component suggests a high level of biological safety of this nanomaterial. As demonstrated in studies *in vitro* and *in vivo*, materials made of h-BN are characterized by lower cytotoxicity, which is relative to different cells, and high biocompatibility. It was shown that BN nanotubes (BNNTs) are not toxic to HEK293 (human embryonic kidney cell line) and CHO (Chinese hamster ovary cell line) cells^8^. A G-chitosan coating used as a wrapping polymer with BNNTs did not affect the viability, metabolic activity, or proliferation of SH-SY5Y cells (human neuroblastoma cell line) at BNNT concentrations higher than 20 µg/ml^11^. Moreover, BNNTs injected up to a dose of 10 mg/kg animal body weight into the rabbit bloodstream did not show any adverse effects in blood, liver and kidney functionality for as long as 7 days after administration^12^. The administration of BNNTs did not change the behaviour or body temperature of the treated rabbits throughout the study period. In contrast, BNNTs affected cellular metabolism in three cancerous cell lines – A549 (adenocarcinoma human alveolar basal epithelial cells), RAW 264.7 (Abelson murine leukaemia virus-induced tumour cells), 3T3-L1 (mouse fibroblast cells), and in normal HEK293 cells. The largest cytotoxic effect of BNNTs was observed in RAW 264.7 cells due to their high endocytic (phagocytic) activity compared to that shown by HEK293 cells. The nanotubes mainly affected the number of RAW 264 cells and their relative viability^13^. Recently, it was shown that highly water dispersible nanostructured h-BN administered at a dose of 2 mg/ml induced higher levels of cytotoxic effects in cancerous MCF-7 (human breast cancer cells) and HeLa (human cervical adenocarcinoma cells) cells than in normal HEK293 cells. Moreover, at lower doses (0.25 mg/ml), h-BN did not affect the morphology of HEK293 cells, whereas increasing the dose of this nanomaterial caused remarkable changes in the cell morphology^1^. These results suggest that, at low doses, BNs are promising nanomaterials suitable for many biomedical applications, including bioimaging, boron neutron capture therapy, drug/peptide/DNA/RNA delivery, and fabrication of advanced implants, *etc*.^1,12^.

The aim of this study was to enhance the hydrophilicity of the h-BN to dispersion it more widely in the aqueous environment and then investigate whether the obtained nanoflakes are cytotoxic. To induce the water solubility of the h-BN nanomaterial, we exfoliated and functionalized bulk h-BN with hydroxyl groups (h-BN-OH). The morphology of the material was examined by transmission electron microscopy, scanning electron microscopy and atomic force microscopy. To confirm the functionalization with hydroxyl groups, spectra obtained from infrared spectroscopy were analysed. The dispersion stability was determined using a UV-Vis spectrometer. Next, using various *in vivo* and *in vitro* methods, we examined the action of the h-BN-OH nanoflakes (h-BN-OH-n) on miscellaneous cellular models: insect haemocytes, human erythrocytes and mouse fibroblasts (the L929 cell line) to detect the possible adverse short- and long-term effects induced by this nanomaterial. We were particularly interested in the effects of h-BN-OH nanoflakes (h-BN-OH-n) in immunocompetent cells during the *in vivo* cellular immune response. As a model for immunoassays, we used the *Tenebrio molitor* beetle because the haemocytes of insects are very sensitive to biotic and abiotic factors. Moreover, haemocytes circulating freely in the open circulatory system of insects exhibit numerous structural and functional similarities to white blood cells responsible for the innate immune response in mammals. The use of haemocytes for the *in vivo* study of the h-BN-OH-n action enabled the detection of any undesirable effects induced by this nanomaterial during the immune response in *T*. *molitor*.

## Results

### The preparation and characterization of nanoparticles

**Figure 1:**
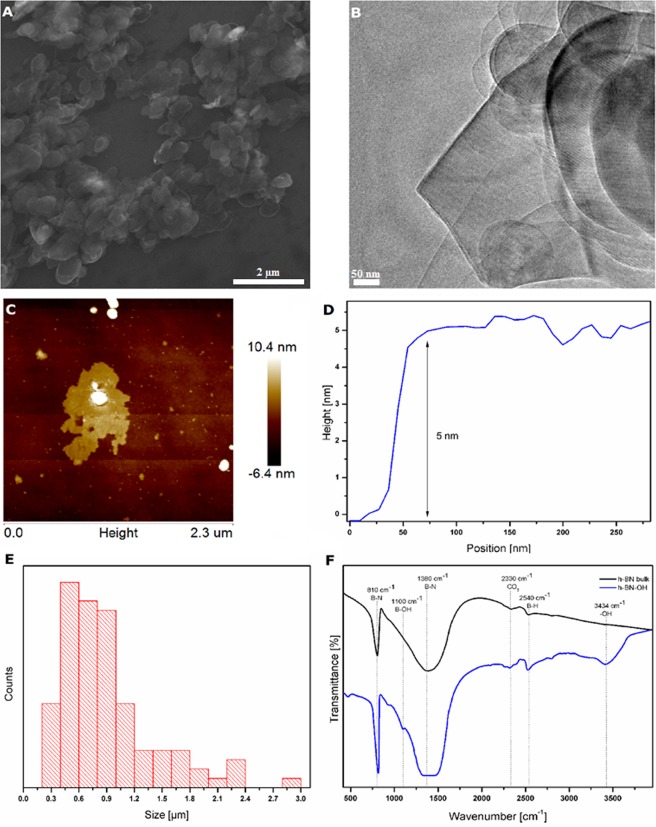
Scanning (**A**) and transmission (**B**) electron microscope images of h-BN-OH, (**C**) AFM of h-BN-OH, (**D**) height profile of h-BN-OH, (**E**) size distribution of h-BN-OH and (**F**) FT-IR spectrum of h-BN-OH and bulk h-BN.

The morphology and thickness of the h-BN-OH are shown in Fig. 1A–C. The flake-like structure of the material is clearly visible. Based on scanning electron microscope images (Fig. 1A), the size distribution of the exfoliated flakes was in the range of 0.3–5 μm with a peak in the 0.4–0.6 μm range (Fig. 1E). This result was confirmed by observations under the atomic force microscope (Fig. 1C). Nevertheless, this material tends to curl/fold/twist similarly to graphene, which enables it to penetrate through the pores of the beetle cuticle. It was also determined that the thickness of the exfoliated flakes was ~5 nm (Fig. 1D), which corresponds to the fifteenth layer of h-BN (the thickness of bulk-h-BN flakes was ~300 nm, corresponding to ~900 layers of individual h-BN).

Fourier transform infrared spectroscopy (FT-IR) was used to confirm the functionalization of h-BN with -OH groups. Figure 1F presents the FT-IR spectra of bulk and functionalized h-BN. There are two characteristic peaks for these materials. The peak at approximately 810 cm^−1^ represents the B–N bending vibration. The peak at 1380 cm^−1^ corresponds to stretching vibrations of B–N^14^. The absorbance peak observed at 2330 cm^−1^ is most likely due to the reaction with carbon dioxide in the atmosphere^15^. The peak at 2540 cm^−1^ was identified as B–H stretching. The confirmation of -OH group formation can be assigned to the presence of the peaks at 3434 cm^−1^ and 1100 cm^−1^, which correspond to -OH groups and to the bonds between the B atoms and the -OH groups, respectively^16^. They are found only in the spectrum of the sample after the functionalization reaction.

**Figure 2:**
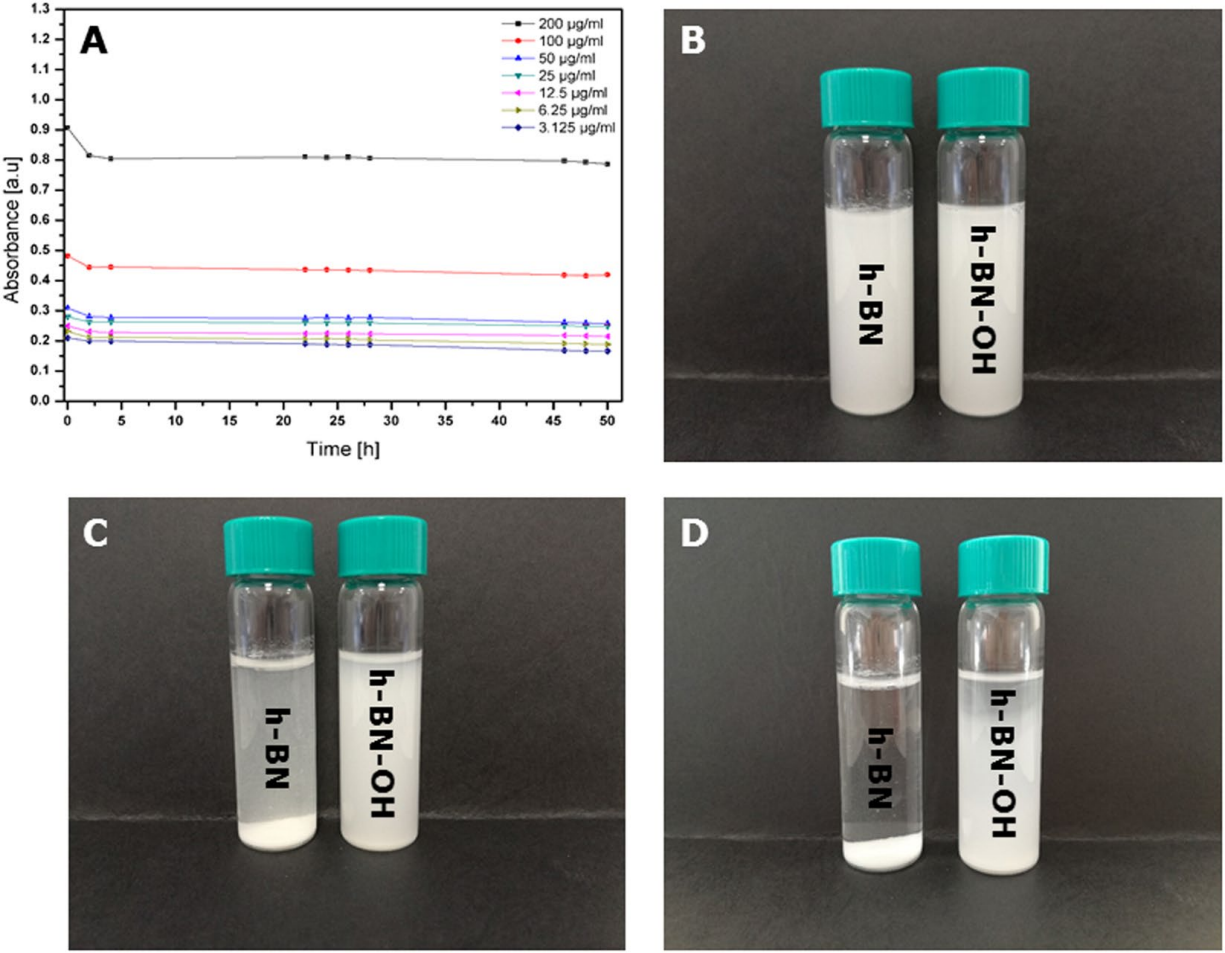
Dispersion stability of h-BN-OH in PBS within the nanomaterial concentration range of 3.125–200 µg/ml (**A**) and photographs of h-BN and h-BN-OH dispersions in PBS with 200 µg/ml nanomaterial after 1 hour of sonication (**B**) and after 24 h (**C**) and 48 hours (**D**) of incubation.

The stability of h-BN-OH is shown in Fig. 2A. UV-Vis spectrophotometer was used to evaluate it. The solution was prepared by dissolving one tablet of PBS in 200 ml of water. The h-BN-OH was added to the PBS solution at concentrations of 3.125, 6.25, 12.5, 25, 50, 100 and 200 µg/ml, respectively. The dispersion stability was monitored for 50 h. In addition, to show the visual differences in the dispersion stability of h-BN and h-BN-OH, digital micrographs were taken. The images show dispersions with a concentration of 200 µg ml^−1^ in PBS after one hour of ultrasonication and after 24 hours and 48 hours of incubation. The dispersion of h-BN-OH is clearly visible even after 48 hours, which was confirmed by optical observation. Moreover, the value of the zeta potential for h-BN-OH was measured to be −19.7 ± 6.81 mV, which means that the h-BN-OH dispersion is stable. Particles with zeta potential values between +20 and −20 mV are usually considered rather unstable, and particles with values that are more positive than −20 mV and more negative than −20 mV are considered stable^17^.

### *In vivo* haemocyte bioassays for determining haemocyte viability, phagocytosis and nodulation

**Figure 3:**
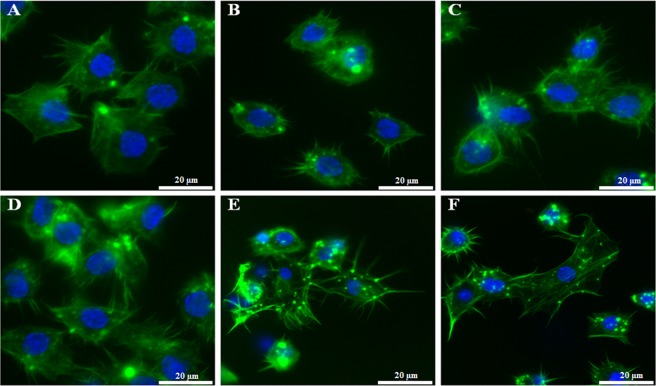
The *in vivo* short- and long-term haemocyte bioassay showing viable, adhesive haemocytes isolated from the insects injected with h-BN-OH-n or topically exposed to h-BN-OH-n. Haemocytes: 2 hours after saline injection (**A**) and 2 days (**D**) after topical application of saline (controls), 2 hours after injection of 2 ng (**B**) or 2 µg (**C**) of h-BN-OH-n, 2 days after topical application of 2 ng (**E**) and 2 µg (**F**) of h-BN-OH-n. The active caspases (1–9) were stained with SR-VAD-FMK (no red  =  no active caspases), the F-actin cytoskeleton was stained with Oregon Green 488 phalloidin (green) and DNA was stained with DAPI (blue). Scale bars: 20 µm.

The haemocytes were viable and did not show changes in morphology. They retained the ability to adhere to the coverslips and to form long filopodia during adhesion (Fig. 3) regardless of the administration route, the dose used, or the time of action of h-BN-OH-n in *T*. *molitor*. SR-VAD-FMK staining showed that h-BN-OH-n did not induce the activation of caspases (1–9) (Fig. 3 and Fig. 5G,H). Oregon Green 488 phalloidin staining demonstrated that these nanoflakes do not cause damage to the cytoskeleton in haemocytes. As shown in Figs 3 and 5G,H, in the control haemocytes and in the haemocytes isolated from the beetle experimental groups, a strong fluorescence signal of the Oregon Green 488 phalloidin was localized subcortically. This regular staining pattern indicates that h-BN-OH-n did not induce depolymerization of the F-actin microfilaments in haemocytes. We also used DAPI staining to visualize changes in chromatin organization. In the nuclei of haemocytes isolated from h-BN-OH nanoflake-exposed beetles, this staining indicated that there had been no DNA fragmentation events.

**Figure 4:**
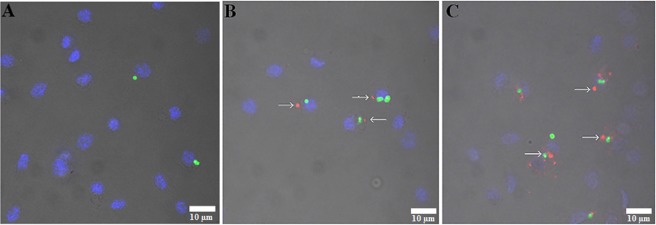
The short-term study of cellular immune response – phagocytosis assay. *Tenebrio molitor* specimens were topically exposed to saline (**A**; control) or 2 ng (**B**) or 2 µg (**C**) of Alexa Fluor 647-h-BN-OH-n and then injected with fluorescent latex beads. Arrows show phagocytes with aggregates of Alexa Fluor 647-h-BN-OH-n (red) and fluorescent latex beads (green). Nuclei of the haemocytes were stained with DAPI (blue). Scale bars: 10 µm.

**Figure 5:**
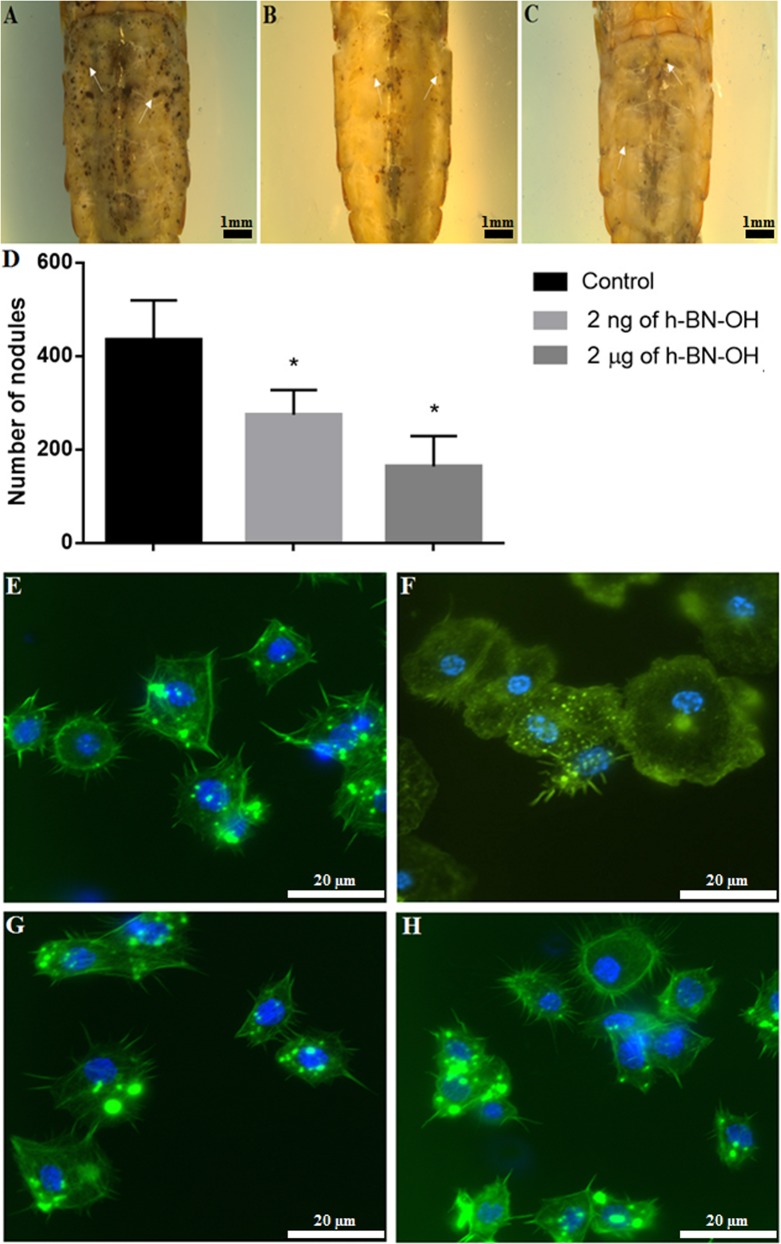
The long-term study of cellular immune response - nodulation and haemocyte viability assay. *Tenebrio molitor* specimens were topically exposed to saline (**A**; control), 2 ng (**B**) or 2 µg (**C**) of h-BN-OH-n. Then, nodulation was induced by injection of a *Staphylococcus aureus* suspension. Arrows show some examples of nodules. Bar: 1 mm. The mean number of nodules formed following the application of saline or h-BN-OH-n (**D**). Values shown are means ± S.D. The results that are significantly different from those of the control group are indicated *p* <0.05 (*). Haemocyte bioassay showing viable, adhesive haemocytes after topical exposure of insects to saline (**E**), saline (**F**), 2 ng (**G**) and 2 μg (**H**) h-BN-OH-n and injection with *Staphylococcus aureus* suspension (**F**,**H**). The active caspases (1–9) were stained with SR-VAD-FMK (no red = no active caspases), the F-actin cytoskeleton was stained with Oregon Green 488 phalloidin (green) and DNA was stained with DAPI (blue). Scale bars: 20 μm.

In haemocytes of beetles topically exposed to Alexa Fluor 647-h-BN-OH-n, strong fluorescence signals were detected two hours and two days after topical application of these nanoflakes (Fig. 4). The fluorescence signals visible in haemocytes indicate that the fluorescent nanoflakes penetrated through the insect cuticle, reached the haemolymph and, finally, were phagocytosed by haemocytes. It can also be seen that the more Alexa Fluor 647-h-BN-OH-n that was applied to the insect cuticle, the more fluorescent nanoflakes were phagocytosed by the haemocytes (Fig. 4B,C). The immunological bioassay also demonstrated that the presence of Alexa Fluor 647-h-BN-OH-n in haemocytes did not impair the ability of these cells to phagocytose the fluorescent latex beads (Fig. 4B,C).

The long-term immunological study showed that h-BN-OH topical application alters the number of nodules formed in the haemocoel of experimentally infected beetles in comparison to the bacterially challenged control beetles (Fig. 5A–D). The nanoparticle-mediated inhibition in nodulation was dose-dependent (Fig. 5D). In insects topically exposed to 2 ng of h-BN-OH-n, the mean number of nodules was 275 (a decrease in nodule formation by 37% compared to those formed in the control); in insects exposed to 2 µg of nanoflakes, the mean number was 164 (a decrease of 67%), while in the control, it was 435 (100% of nodules formed) (Fig. 5D). In this bioassay, we also observed the morphology, viability and adhesion of haemocytes on the third day after the insects were topically exposed to h-BN-OH-n and the bacteria challenge. As shown in Fig. 5E–H, the haemocytes of all studied groups were viable. The control haemocytes that were taken from the bacteria-unchallenged insects formed long filopodia upon adhering to coverslips and were evenly spread on coverslips (Fig. 5E). In response to *Staphylococcus aureus* infection, the haemocytes exhibited a greater ability to adhere to coverslip than control haemocytes, where they formed microaggregates and short and delicate filopodia (Fig. 5F). The haemocytes of beetles that were topically exposed to 2 ng or 2 µg of h-BN-OH-n and then infected with bacteria did not aggregate and did not exhibit a greater ability to adhere to coverslips in response to bacterial infection (Fig. 5G,H). These haemocytes had the same morphology and adhesion as the haemocytes taken from the control insects that had not been infected with bacteria.

### RBC biocompatibility assays

#### Changes in RBC morphology and cell membrane permeability

**Figure 6:**
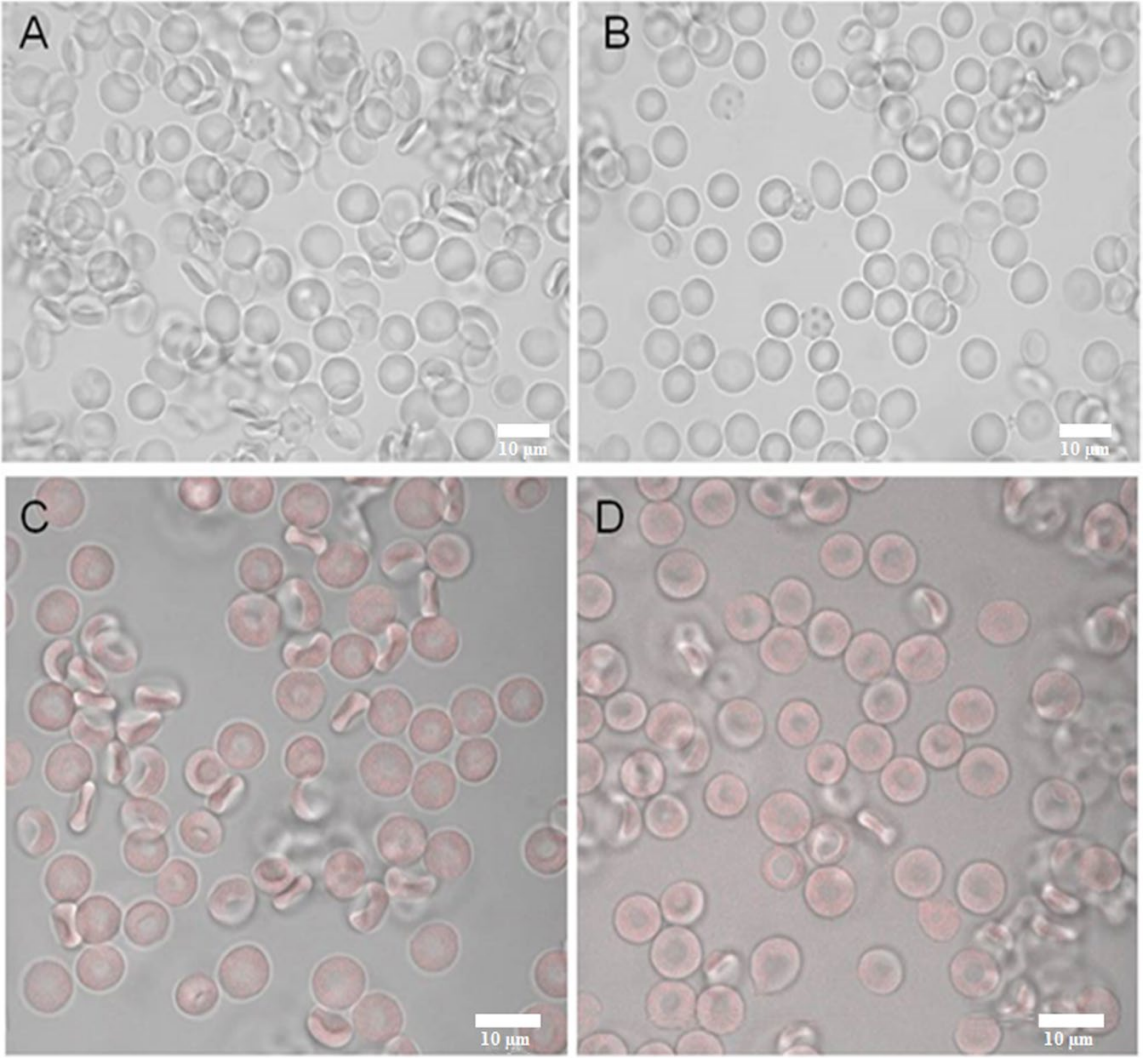
The effect of h-BN-OH on RBC morphology (1 hour at 37 °C) as observed with light microscopy: (**A**) control cells (PBS buffer) and (**B**) cells incubated with h-BN-OH-n (10^−7^ g/ml). The binding effect of Alexa Fluor 647-h-BN-OH-n on RBCs as observed by confocal microscopy: (**C**) control cell (PBS buffer) and (**D**) cells incubated with h-BN-OH-n (10^−7^ g/ml). Scale bars: 10 µm.

The results obtained for all h-BN-OH-n concentrations used and different incubation times are summarized in Table 1. After a brief incubation (1 hour and 4 hours, 37 °C), h-BN-OH-n had no significant effect on the RBC discocytic shape (Fig. 6A,B) or membrane permeability (Table 1, haemolytic activity <5%) for doses administered in the concentration range. After a long-term incubation (24 hours, 37 °C), h-BN-OH-n at 10^−7^ g/ml and at 10^−8^ g/ml induced weak haemolysis (>5%). Weak echinocytosis (disco-echinocytes) was observed for both the control and h-BN-OH-n-incubated cells.

**Table 1:** The effect of the h-BN-OH nanoflakes on RBCs after different incubation times (1 hour, 4 hours, and 24 hours at 37 °C) at the given concentration. Haemolysis above 5% indicates weak haemolytic activity

Concentration/Incubation condition	10^−7^ g/ml		10^−8^ g/ml		10^−9^ g/ml	
	Haemolytic activity (%)	Dominate RBC shape	Haemolytic activity (%)	Dominate RBC shape	Haemolytic activity (%)	Dominate RBC shape
**1 h**, **37** °**C**						
Control (PBS)	1.44 ± 0.52	D	1.72 ± 0.36	D	1.49 ± 0.73	D
h-BN-OH	3.78 ± 0.86	D	2.73 ± 1.25	D	1.91 ± 1.24	D
**4 h**, **37** °**C**						
Control (PBS)	1.61 ± 0.68	D	1.97 ± 0.83	D	2.07 ± 0.49	D
h-BN-OH	4.18 ± 0.39	D	3.08 ± 0.64	D	2.19 ± 1.25	D
**24 h**, **37** °**C**						
Control (PBS)	3.81 ± 0.72	D/DE	3.32 ± 1.16	D/DE	3.25 ± 0.63	D/DE
h-BN-OH	8.16 ± 1.09	D/DE	6.85 ± 1.64	D/DE	4.22 ± 0.35	D/DE

The presented values are the mean ± SD of three independent experiments with RBCs from different donors; abbreviations: D – discocytes (as control cells), DE – discoechinocytes.

#### Detection of the membrane-bound h-BN-OH nanoflakes using confocal microscopy

Alexa Fluor 647-h-BN-OH-n at the highest concentration (10^−7^ g/ml) did not bind the RBC membrane at the levels detectable by confocal microscopy (Fig. 6C,D). No noticeable changes were observed between the autofluorescence of the control erythrocytes and the RBCs exposed to the nanoflakes (Fig. 6C,D).

#### Erythrocyte sedimentation rate with nanoflakes

**Figure 7:**
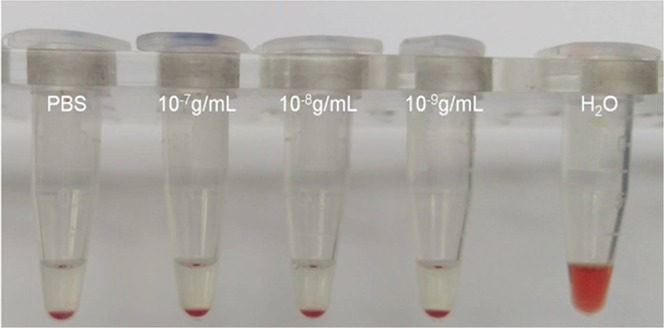
The erythrocyte sedimentation rate in the presence of h-BN-OH-n. The concentration of h-BN-OH-n is indicated on the EP vials. PBS – control erythrocytes.

RBCs incubated with h-BN-OH-n at concentrations ranging from 10^−7^ to 10^−9^ g/ml settled at the same sedimentation rate as the control RBCs incubated in PBS only (Fig. 7).

#### Haemoglobin oxidation

Methaemoglobin levels were assayed after 1 hour incubation at 37 °C in the presence of the highest concentration of h-BN-OH-n (10^−7^ g/ml), resulting in 1.949 ± 0,065% for control RBCs and 1.789 ± 0,093% for RBCs incubated with h-BN-OH-n. The difference in the methaemoglobin level between the control RBCs and the RBCs incubated with h-BN-OH-n was not statistically significant (*p* > 0.05). Our results indicated that h-BN-OH nanoparticles did not enhance haemoglobin oxidation or methaemoglobin formation. Because of possible nanoparticle aggregation in the solution that could affect their biological activity, the incubation time was restricted by to no more than 1 hour.

#### ROS formation

**Figure 8:**
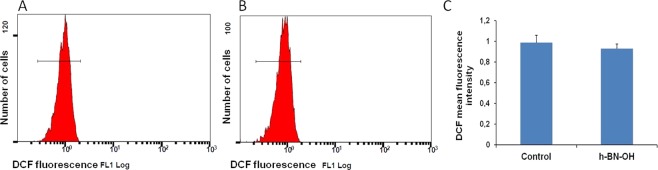
Effect of h-BN-OH-n on ROS formation in human erythrocytes. Histogram of DCF fluorescence in RBCs following exposure for 1 hour to (**A**) PBS and (**B**) h-BN-OH-n (10^−7^ g/ml). (**C**) Mean fluorescence intensity (±SD, n = 5) of the DCF in RBCs exposed to PBS (control) and h-BN-OH-n.

To investigate the effect of h-BN-OH-n on oxidative stress in intact RBCs, the intracellular ROS level was estimated utilizing 2′,7′-dichlorodihydrofluorescein diacetate (DCF-DA) (Fig. 8A,B). The exposure to the highest concentration (10^−7^ g/ml) of h-BN-OH-n for a short time (1 hour) did not increase the DCF fluorescence (Fig. 8C). The mean fluorescence intensity for the control cell was equal to 0.986 ± 0.0740, and the mean fluorescence intensity for the h-BH-OH-treated cell was equal to 0.930 ± 0.0447 (*p* > 0.05). These results showed that h-BN-OH-n did not enhance the ROS level in the RBCs.

### The study of the relative viability of the L929 cells

**Figure 9:**
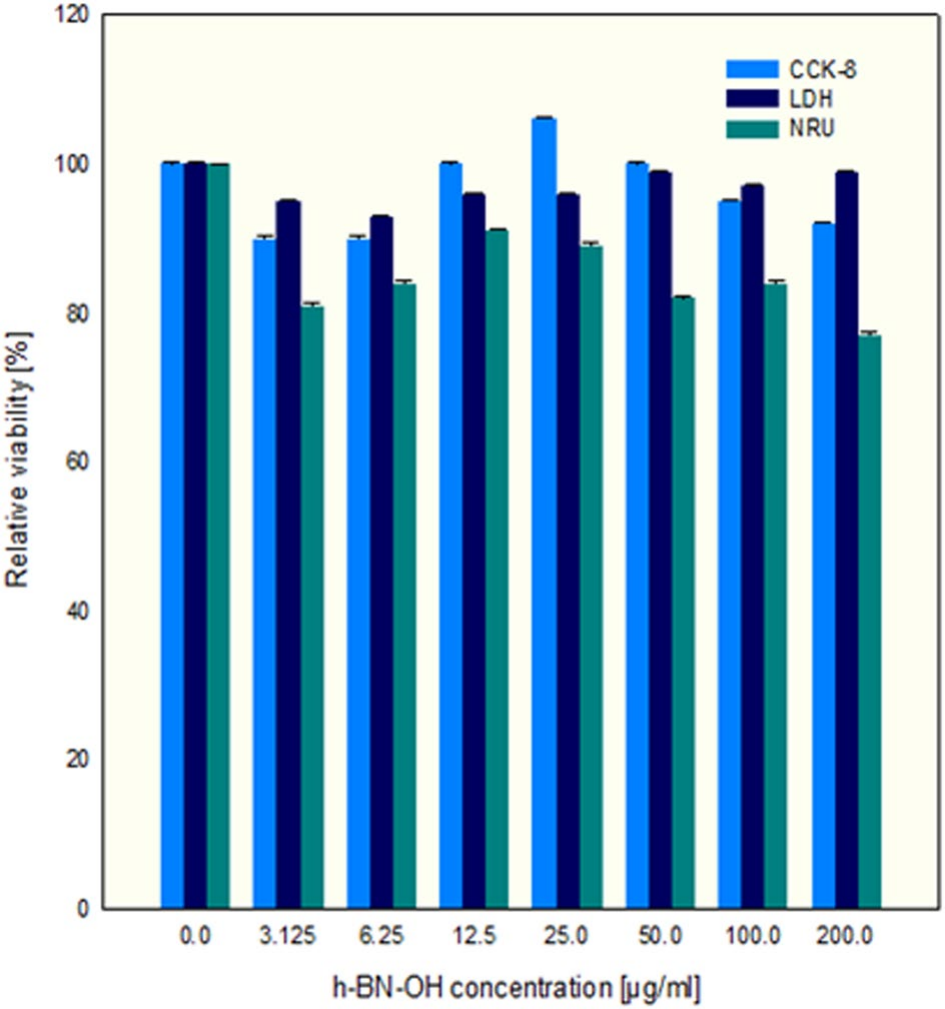
Relative viability of L929 cells after 24-hour incubation with h-BN-OH-n. Values shown are means ± S.D.

The biocompatibility of h-BN-OH-n at concentrations of 3.125 to 200.0 µg/ml was determined using CCK-8, LDH and NRU assays. The CCK-8 assay showed the highest reduction (approximately 10%) in mitochondrial activity in the h-BN-OH-treated L929 cells at a concentration of h-BN-OH-n of 3.125 and 6.25 μg/ml compared to activity in the control. The nanoflakes at concentrations of 100.0 µg/ml and 200.0 µg/ml caused a slight reduction in mitochondrial metabolism in these cells (5% and 8% for concentrations of h-BN-OH-n of 100.0 and 200.0 µg/ml, respectively) in comparison to the level in the control cultures (Fig. 9). The membrane integrity assay showed that LDH leakage from L929 cells incubated in h-BN-OH-n solution at concentrations of 3.125 and 6.25 µg of nanoparticle/ml increased by 7% and by 5%, respectively, compared to that from the control. The other studied concentrations of h-BN-OH-n affected the membrane integrity in the range of 1–4% (Fig. 9). The NRU assay showed that the relative viability of L929 cells at concentrations of 3.125 and 200.0 µg of h-BN-OH/ml was the lowest and reached approximately 81 and 77% compared to that of the control. At other doses of the nanomaterial (6.25–100.0 µg/ml), the viability of L929 cells decreased to 82–91% (Fig. 9). Statistical analysis of all test results did not reveal any significant differences between any of the experimental and the control cultures (*p* > 0.05).

## Discussion

This study shows that the functionalization of the h-BN material with hydroxyl groups significantly increased its hydrophilicity and, as a result, enabled h-BN-OH to disperse stably in aqueous solution. The acquired effect is consistent with that previous reports in which this type of surface functionalization has been applied^18^. Because of the strong B–N (2pπ-2pπ) bonds that envelope the outer surface of the h-BN, the h-BN does not react to moisture and is hydrophobic. Introducing the hydroxide functional groups on the h-BN surface not only increases the stability of the dispersion but also enables the modification by grafting more complex functional groups/molecules to alter its surface chemical/physical properties. A summary of the possible functionalities and changes that h-BN undergoes during hydroxyl group functionalization has been presented by Zheng *et al*.^19^.

RBCs are the most abundant cells in human blood and act as oxygen transporters^20^. These cells are devoid of a nucleus and other organelles; therefore, they are used as a simple model to screen induced toxicity of different compounds, including nanoparticles^21–24^. The main determinants of cytotoxicity to RBCs are (i) changes in discoid shape, as an effect of the interaction of compounds with the components of egzoplasmic and endoplasmic leaflets of the cell membrane, and (ii) haemolysis, as an effect of significant disturbance of the molecular structure of the membrane because of egzogenic molecule incorporation^20,25^.

We used RBCs to investigate the interaction of h-BN-OH-n with the cell membrane and with haemoglobin, which is the most important RBC protein. Haemolysis is defined as the destruction of the RBC membrane, which can lead to anaemia *in vivo*^26^. One type of haemolysis is oxidative haemolysis, which is dependent on the detrimental effects of ROS^27^. Oxidative stress can be generated by nanoparticles^28^; therefore, the mechanism by which some nanoparticles exert cytotoxicity is related to ROS induction, methaemoglobin formation and oxidative cell damage. In this study, after brief incubation with h-BN-OH-n, neither significant erythrocyte shape transformation (Fig. 6) nor significant haemolysis (Table 1) was observed. Weak haemolysis (8.16% ± 1.09) was detected after long-term incubation at the highest concentration of h-BN-OH-n. However, this concentration (10^−7^ g/ml) is above any possible level applied under *in vivo* conditions. This result can be explained by self-nanoparticle aggregate formation acting as the potential membrane-perturbing agents, as we have observed for other nanoparticle types^21,24^. However, after short-term incubation, h-BN-OH-n did not form self-aggregates that were detectable by light microscopy (Fig. 6B,D) and did not influence the RBC sedimentation rate (Fig. 7). Moreover, no statistically significant increase in methaemoglobin or the ROS levels was observed in the RBCs (Fig. 8). It can be concluded that h-BN-OH-n did not affect human RBC structure or function after short-term incubation *in vitro*. These results are in agreement with *in vitro* results obtained with rat erythrocytes^5^.

Many different data indicate that BN nanomaterials are nontoxic or induce very low levels of cytotoxicity in various mammalian cell lines^11,12,29–33^. For this reason, chemically functionalized BNs are of interest as carriers of anticancer drugs. It has been shown that BN nanospheres coupled with folate or poly(allylamine)-citraconic anhydride (PAH-cit) are biocompatible up to a concentration of 100 μg/ml and can serve as an excellent delivery system for doxorubicin hydrochloride (DOX), a commonly used anticancer drug, to cancer cells. These complexes effectively release DOX at low pH, to induce cytotoxicity in HeLa cells; therefore, this nanomaterial has strong potential for targeted cancer therapy^34,35^. In this study, we confirmed the high biocompatibility of h-BN-OH-n (in the range of concentrations tested) with L929 cells using cytotoxic tests, providing a quantitative estimation of the number of viable cells in cell culture. We observed a slight decrease in the viability of L929 cells as induced by h-BN-OH-n (Fig. 9). Most likely, at lower concentrations of h-BN-OH-n, these nanoparticles did not aggregate and could freely penetrate the cell membrane. Inside the cell, they could affect the mitochondrial and lysosomal membranes, negatively affecting their integrity. As a result of the impaired integrity of these organelles, their functions could be disrupted, and as a consequence, the cells died. On the other hand, during the 24-hour incubation of L929 cells with h-BN-OH-n at the highest concentration, these nanoparticles may have formed aggregates in the cell culture medium. These aggregates could interact with the cellular membrane of human RBCs and L929 cells, causing cell membrane dysfunction and damage to these cells, as we observed for other types of nanoparticles^21,24^.

In contrast to the non-adhesive red blood cells circulating in capillaries in vertebrates, circulating insect haemocytes exhibit a change in morphology and behaviour, enabling an effective fight against pathogens, such that they resemble mammalian leucocytes, e.g., macrophages, dendritic cells and neutrophils^36^. In insects, these cells play a key role in the immune response, including cellular defence (phagocytosis, nodulation, encapsulation and clotting) and humoural defence (phenoloxidase activity)^37,38^. During various immune challenges, insect haemocytes quickly and efficiently phagocytose quickly and efficiently foreign targets, such as biotic-like bacteria, yeast and viruses and abiotic-like synthetic beads or particles of India ink^39–41^. In this study, we used *in vivo* haemocyte bioassays to show that nanoflakes of h-BN-OH penetrated the cuticle to access the body of the insect, despite the unique structure of the cuticle that protects the insect against the effects of various unfavourable factors^42^. We showed in a short- and long-term experiments that h-BN-OH-n injected into the haemolymph or applied on the cuticle of *T*. *molitor* at the studied doses did not change the haemocyte morphology and did not induce cytotoxic effects that result in apoptosis (Figs 3, 5E–H). The *in vivo* analysis of h-BN-OH-n passage through the insect cuticle demonstrated that Alexa Fluor 647-h-BN-OH-n molecules were phagocytosed by haemocytes and were aggregated inside these cells. This result shows that h-BN-OH nanoflakes breached the tight, hydrophobic barrier of the insect cuticle despite the increased hydrophilicity of h-BN-OH. In the cuticle of *T*. *molitor* beetle, there are lipid-coated nanopores with diameters of 6–65 nm that enable the mealworm to detect and secrete pheromones or other physiological substances^43,44^. This specific organization of the insect cuticle limits the size of the molecules that can penetrate to the interior of insects through the transcuticular pathway^42^. The presence of the fluorescent h-BN-OH aggregates detectable by confocal microscopy in haemocytes shows that the cuticle serves as a sieve for nanoflakes because only small nanoparticles can penetrate through the nanopores of the cuticle to enter the insect’s body, where they are immediately phagocytized after reaching the haemolymph^42^. Moreover, the presence of nanoflakes aggregates in the haemocytes after the insect is topically exposed to Alexa Fluor 647-h-BN-OH confirmed the effective increase in the hydrophilic properties of this nanomaterial as a result of its functionalization with hydroxyl groups. As shown in Fig. 3, the increased hydrophilicity of this nanomaterial compound enabled a stable dispersion of h-BN-OH-n in an aqueous solution after sonication, which was a prerequisite for the bioavailability of h-BN-OH through topical exposure of the insect. It should be emphasized that despite the increased hydrophilicity of the nanomaterial, it can pass through the hydrophobic nanopores in the insect cuticle. This fact may indicate the amphipathicity of this nanomaterial after functionalization.

The phagocytosis study also showed that h-BN-OH-n did not affect the haemocyte cell membrane, and the dose-dependent presence of the fluorescent h-BN-OH-n in haemocytes did not interfere with the ability of these cells to phagocytose much larger abiotic particles, such as latex beads (Fig. 4). This result suggests that the short-term action of h-BN-OH-n in the doses used in this study did not change haemocyte viability and did not impair the complex function of the haemocyte cell membrane, which is responsible for recognition of invading pathogens or abiotic targets and their efficient engulfment by the haemocyte. However, questions arise: How are abiotic targets such as nanoparticles recognized by haemocytes, what signalling molecules regulate phagosome formation and what is the fate of the nanoparticles in the phagocytes? Despite the lack of answers to these questions, the above experiments may support the validity of the statement that the h-BN-OH-n nanoparticles are biocompatible with insect haemocytes, as in the case of human erythrocytes and mouse L929 cells. However, the study of nodulation, another type of cellular immune response in insects, indicates the need for caution in formulating such an opinion.

Nodulation is the most important cellular immune defence mechanism against a large number of bacteria, fungi and viruses that infect insects^36,37^. Upon pathogen infection, the target molecules of invading microorganisms or parasites are recognized by the haemocytes, and the formation of nodules or capsules begins. Nodulation refers to the complex process that is initiated by the activation of haemocytes circulating in haemolymph, where they change from non-adhesive to adhesive cells with a tendency to form aggregates surrounding the pathogens. The formed nodules eliminate invading microorganisms from insect’s circulation^36^. Our previous long-term study in *T*. *molitor* showed that injection of an insect peptide, alloferon, and then, *S*. *aureus* infection increased haemocyte adhesion, leading to rearrangement at the periphery of the cell and resulting in a separation of the platelet-like fragments of haemocyte that retained the ability to adhere and to form filopodia^45^. These changes in the morphology and behaviour of haemocytes enable them to dispose of bacteria efficiently through the formation of numerous nodules around aggregates of bacteria^45^. The current long-term *in vivo* experiment showed that haemocytes isolated from the insects exposed to nanoflakes and then challenged with bacteria (Fig. 5G,H) were viable, as were the haemocytes of the bacteria-challenged insects, but they had different morphology and did not exhibit an enhanced ability to adhere to coverslips. Their morphology and adhesion ability were the same as those of control haemocytes (Fig. 5E,G,H). This result indicates that, after exposure to h-BN-OH nanoflakes, the haemocytes could not be activated in response to an experimental bacterial infection. Simultaneously, potent nanoparticle-mediated inhibition of nodule formation was observed in the haemocoel of insects after bacterial challenge (Fig. 5A–D). Taken together, these results suggest that a reduction in nodule formation upon the action of h-BN-OH-n may result from disrupted cell behaviour, including a decreased ability of the circulating haemocytes to recognize bacteria, migrate towards them or microaggregate around them. There are indications that phagocytosis and nodulation are regulated by eicosanoids synthesized in the fat body. Eicosanoids increase the ability of haemocytes to phagocytose, migrate, adhere, elongate, microaggregate and spread during nodulation and enable the release of phenoloxidase from haemocytes to kill pathogens within the forming nodules^46^. We suggest that the decrease in nodulation may be caused by the nanoparticle-mediated inhibition of eicosanoid synthesis in the insect’s fat body, which in turn may lead to impaired haemocyte function and dysfunctions of the immune system. It may be suggested that the short-term exposure of insects to h-BN-OH-n did not affect eicosanoid synthesis because, upon nanoparticle action, the haemocytes retained the ability to phagocytose nanoparticles and latex beads. However, to clarify these issues, further detailed studies on the time-dependent influence of h-BN-OH-n on eicosanoid synthesis in the fat body of insects is required.

## Conclusions

This study shows that the functionalization of the h-BN by hydroxyl groups significantly increased the hydrophilicity of this material, enabling it to disperse in aqueous solutions in the form of nanoflakes. We proved that the nanoflake dispersion was stable and that the size of h-BN-OH-n enabled it to penetrate through such a tight biological barrier as the beetle’s cuticle. Our study also showed that a mode of the h-BN-OH-n action on cells may vary depending on the species, type of cells tested, their function, and duration of nanoparticle exposure of the cells. The *in vivo* study on insect haemocytes and the *in vitro* study on mouse L929 cells and human erythrocytes showed that this nanomaterial confers low cytotoxicity, which suggests its poor reactivity with biological systems. However, a long-term study in *T*. *molitor* has shown that h-BN-OH-n, despite low cytotoxicity, can significantly affect the behaviour of immunocompetent cells and ultimately their function during the immune response. This work does not unambiguously confirm that h-BN-OH-n is harmless to the health of animals and humans. Knowledge about the distinct impact of h-BN-OH-n on various living organisms is therefore indispensable for the future practical application of this nanomaterial in biomedicine. Therefore, it is necessary to study *in vivo* the long-term effects of h-BN-OH-n in various animal models to ultimately decide whether this nanomaterial is safe and does not cause unexpected reactions.

## Methods

### Exfoliation, -OH functionalization and fluorescent dye labelling of h-BN

The exfoliation and –OH functionalization was performed in simple steps: (1) 750 mg of bulk h-BN (Sigma Aldrich) was introduced in a three-necked flask with 3 g of KMnO_4_ and 60 ml of H_2_SO_4_. The mixture was refluxed at 40 °C for 6 hours. After this time, the resulting material was centrifuged and washed with water until the pH was neutral. The powder was dried at 50 °C overnight. In the next step (2), 50 mg of h-BN was placed in a three-necked flask with 200 ml of hydrogen peroxide (30%, Sigma Aldrich). The mixture was refluxed for 48 h at 110 °C. Finally, the material was centrifuged (10 min at 10000 rpm), washed twice with water and dried overnight at 50 °C.

To show that the h-BN-OH nanoflakes can penetrate through the cuticle of the insect and enter the haemocoel, we labelled h-BN-OH with a fluorescent dye. A solution of 1 µg/ml Alexa Fluor 647 (Thermo Fisher Scientific) in dimethylformamide (DMF, Sigma Aldrich) was prepared. Ten milligrams of h-BN-OH was mixed with 10 ml of Alexa Fluor 647. After 24 hours, the material was centrifuged (5 min at 5000 rpm), washed with DMF and water, and dried overnight at 35 °C.

The h-BN-OH or the Alexa Fluor 647-h-BN-OH stock solution was prepared by sonication with 1 mg of the appropriate powder in 1 ml of ultra-pure distilled water for 4 h. The working solution was prepared in physiological saline from freshly sonicated stock solution immediately before application. The samples were examined using transmission electron microscopy (TEM, FEI Tecnai F30, Frequency Electronics Inc.) and by scanning electron microscopy (TESCAN, VEGA SBU3). The topography of the flakes was measured by atomic force microscopy (AFM, Nanoscope V Multimode 8, Bruker). The presence of the –OH functional groups was confirmed by FT-IR spectroscopy (Nicolet 6700 FT-IR spectrometer from Thermo Scientific). The stability of the h-BN water-based suspension was indicated via zeta potential measurements performed in a Zeta Sizer (ZS Nano, Malvern) and by UV-Vis monitoring (Thermo Scientific GENESYS 10S, Thermo Fisher Scientific, Waltham, MA, USA).

The dispersion stability of the hexagonal boron nitride functionalized with OH groups was examined in phosphate buffered saline (PBS). Subsequently, different amounts of nanomaterial were diluted with PBS and sonicated to obtain a homogeneous solution of the following concentration 3.125, 6.25, 12.5, 25, 50, 100 and 200 µg of h-BN-OH/ml, respectively. The UV-Vis monitoring (Thermo Scientific GENESYS 10S, Thermo Fisher Scientific, Waltham, MA, USA) at 450 nm was used to determine the dispersion stability at the selected time intervals (from 1 to 50 hours).

### Insects

*T*. *molitor* L. were reared as described previously^45^. All insects in our experiments were derived from parents that were less than 1 month old. Studies were carried out on fifteen 4-day-old adult beetles for each treatment. During all days of exposure to the h-BN-OH nanoflakes, we did not observe significant differences in the behaviour of the beetles or significant increases in the mortality rate compared with that of the control group.

#### *In vivo* haemocyte bioassays

Short- and long-term study of haemocyte viability after injection of h-BN-OH-n or topical exposure to h-BN-OH-n: The cytotoxicity of h-BN-OH has been evaluated on insect haemocytes using a haemocyte bioassay *in vivo* as previously described^47^. Injection of the test compound was performed to understand the rapid effect of h-BN-OH on haemocytes by an established procedure^47^, whereas a topical application assay was conducted to understand the long-term effect of the compound. Adult beetles were split into six experimental groups—including two saline-treated controls group and fourth-BN-OH-treated groups. The first and second experimental groups of beetles were injected with the h-BN-OH-n solution (2 µl) at a dose of 2 ng or 2 µg of nanoflakes per insect using a Hamilton syringe (Hamilton Co., Bonaduz, Switzerland), whereas each insect in the third and fourth experimental beetle groups of beetles received 2 ng or 2 µg of nanoflakes in h-BN-OH-n solution (2 µl) administered topically to the ventral side of thorax. The control group of beetles was injected with the same volume of saline or was exposed to saline topically. Two hours after injection and two days after topical application of nanoflakes, haemolymph samples (5 µl) were collected; the haemocytes were prepared and used for the appropriate microscopic studies. The haemocytes were stained for the detection of active caspase using an inhibitor of caspase (1–9) activity (a sulforhodamine derivative of the valyl-alanyl-aspartic acid fluoromethyl ketone, SR-VAD-FMK; AK-115, BIOMOL, Plymouth Meeting, PA, USA), for detection of F-actin cytoskeleton using Oregon Green 488 phalloidin (Invitrogen, Thermo Fisher Scientific, MA, USA) and for visualization of haemocyte nuclei using DAPI^47^. The haemocytes were examined with a Nikon Eclipse TE 2000-U fluorescence microscope to study the haemocyte morphology, adhesion and viability.

#### Short-term immune response - phagocytosis

To test whether h-BN-OH-n can penetrate through the cuticle into the insect haemocoel, an *in vivo* phagocytosis bioassay was used. Simultaneously with the same biotest, we studied whether, after phagocytizing the h-BN-OH nanoflakes, the haemocytes retained the ability to phagocytose another abiotic target – the fluorescent latex beads. Briefly, adult beetles were split into three experimental groups—including the saline-treated control group and two Alexa Fluor 647-h-BN-OH-treated groups. All insects were anaesthetized, and the control group was topically exposed to physiological saline (2 µl). The second group was topically exposed to Alexa Fluor 647-h-BN-OH-n solution (2 µl) in a dose of 2 ng per insect, whereas the third group received 2 µg of Alexa Fluor 647 solution (2 µl) topically per insect. Four hours after saline and nanoflake exposure, the insects were anaesthetized again, disinfected and injected with 2 µl of the fluorescent latex bead suspension (diluted at a ratio of 1:1000 v/v in sterile saline). Haemolymph samples (5 µl) were collected one hour after the latex bead injection, and the haemocytes were prepared^47^. The haemocytes were washed with saline, fixed with 4% paraformaldehyde for 10 min and stained with DAPI solution to visualize nuclei. Then, the haemocytes were washed again, mounted and examined with a Zeiss LSM 510 confocal microscope to detect the Alexa Fluor 647-h-BN-OH nanoflakes and fluorescent latex beads in the haemocytes.

#### Long-term immune response - nodulation and the *in vivo* haemocyte viability bioassay

The nodule formation after exposing beetles to h-BN-OH-n was studied in male beetles after bacterial challenge according to the method previously described^45^. Simultaneously, these beetles were also tested using an *in vivo* haemocyte viability biotest to investigate whether h-BN-OH-n induced long-term haemocytotoxic effects. For this purpose, insects were split into three groups and subsequently anaesthetized and topically exposed to saline and h-BN-OH-n nanoflake solutions as described above. Two hours after saline or h-BN-OH-n exposure (in a dose of 2 ng or 2 µg of h-BN-OH per insect), the beetles were anaesthetized again, disinfected, washed in distilled water, and injected with 0.05% *S*. *aureus* solution in saline (2 μl, formalin-fixed suspension of non-viable *S*. *aureus*; Sigma® 107 S2014). Three days after the bacterial injection, the haemolymph samples (5 µl) were first collected to study the viability and the adhesion of the haemocytes as described above, and then, nodule formation was determined. The beetles were dissected to expose the nodules on the dorsal side of the haemocoel. The insect body was cleaned to remove the fat body, Malpighian tubules and alimentary canal. The nodules were counted on the dorsal side of the beetle under an Olympus SZX 12 stereoscopic microscope, and three images of each insect were taken with an Olympus U-LH100HG digital camera. Images were analysed with the ImageJ (version 2) computer program.

### *In vitro* human erythrocyte studies

#### Erythrocyte preparation

Fresh red blood cell (RBC) suspensions were acquired for a fee from the blood bank and washed three times (3000 rpm for 10 min at 4 °C) in 7.4 pH phosphate buffered saline (PBS – 137 mM NaCl, 2.7 mM KCl, 10 mM NaHPO_4_, and 1.76 mM KH_2_PO_4_) supplemented with 10 mM glucose. After washing, the cells were suspended in PBS buffer at 1.65 × 10^9^ cells/ml, stored at 4 °C and used within 5 hours.

#### Haemolysis

A haemolysis assay was performed as described previously^27^. RBCs (1.65 × 10^8^ cells/ml, 1.5% haematocrit) were incubated in PBS buffer without and with h-BN-OH-n in the range of concentrations of 10^−7^–10^−9^ g/ml for 1 hour, 4 and 24 hours at 37 °C in a shaking water bath. The stock solution of h-BN-OH-n was prepared in PBS. Samples with RBCs incubated in PBS buffer were taken as the controls. Each sample was repeated three times, and the experiments were repeated three times with RBCs from different donors. The degree of haemolysis was estimated by measuring the absorbance of the supernatant at 540 nm in an EPOLL 2000 ECO spectrophotometer (PZ EMCO, Warsaw, Poland).

#### Erythrocyte shape transformation and nanoparticle binding

Following the incubation protocol of RBCs and h-BN-OH or Alexa Fluor 647-h-BN-OH nanoflakes as described above, cells were fixed in 5% paraformaldehyde (PFA) plus 0.01% glutaraldehyde (GA) for 1 hour at room temperature. Fixed RBCs were washed by exchanging the supernatant with PBS buffer, settled on poly-L-lysine-treated (0.1 mg/ml, 10 min, RT) coverslips and mounted with 80% glycerol. The coverslips were sealed with nail polish. Many RBCs from several separate experimental samples were studied using a Zeiss LSM 510 confocal microscope.

#### Erythrocyte sedimentation rate (ESR)

The ESR was determined as described previously^21^. RBCs (1.65 × 10^8^ cells/ml) were incubated with 10^−7^ g/ml, 10^−8^ g/ml or 10^−9^ g/ml of h-BN-OH-n for 1 hour at 37 °C. The erythrocytes incubated in PBS were used as the control. Each sample was prepared in triplicate, and the experiments were repeated three times.

#### Methaemoglobin assay

The haemoglobin oxidation was determined as the methaemoglobin level according to the method described previously^48^. Briefly, after incubation of RBCs with 10^−7^ g/ml of h-BN-OH-n for 1 hour at 37 °C, cells were treated with deionized water (+4 °C for 2 hours) and centrifuged (3000 rpm for 10 min at 4 °C). The absorbance of the supernatants was measured at λ = 630 nm and λ = 700 nm to determine the level of methaemoglobin. After measurements, potassium ferricyanide (K_3_[Fe(CN)_6_]) was added to the samples, and they were re-assayed for absorbance at the same wavelengths (positive control). The percentage of methaemoglobin was calculated by the following equation (1):

1 $${\rm{MetHb}}[ \% ]={{\rm{A}}}_{630}-{{\rm{A}}}_{700}/{{\rm{A}}}_{100 \% {\rm{metHb}}630}-{{\rm{A}}}_{100 \% {\rm{metHb}}700}$$

where MetHb [%] – the percentage of haemoglobin

A630 – the absorbance of the sample tested at λ = 630 nm

A700 – the absorbance of the sample tested at λ = 700 nm

A100% metHb630 – the absorbance of the sample treated with K_3_[Fe(CN)_6_] at λ = 630 nm

A100% metHb700 – the absorbance in the sample treated with K_3_[Fe(CN)_6_] at λ = 700 nm

#### Reactive oxidant species (ROS) production

The oxidative stress inside intact RBCs was determined by the oxidant-sensing fluorescent probe 2′,7’-dichlorodihydrofluorescein diacetate (DCF-DA, Sigma D6883, Poznań, Poland) as described previously^49^. Briefly, after incubation of RBCs with 10^−7^ g/ml of h-BN-OH-n for 1 hour at 37 °C, 100 μl of suspended RBCs was washed in PBS and then stained with DCF-DA at a final concentration of 10 μM at 37 °C for 30 min in the dark and washed in PBS. The DCF-DA-loaded RBCs were re-suspended in 1000 μl PBS, and the ROS-dependent mean fluorescence intensity was measured (10 000 cells) at an excitation wavelength of 488 nm and an emission wavelength of 530 nm on a Cytomix FC 500 MPL flow cytometer (Beckman Coulter).

### L929 cell studies *in vitro*

#### Lactate dehydrogenase leaking assay

The effect of h-BN-OH-n on the membrane integrity of L929 cells was evaluated using an LDH CytoTox 96^®^ non-radioactive cytotoxicity assay (Promega, Madison, WI, USA). The assay was performed according to the manufacturer’s instructions, and the absorbance was measured at 490 nm using a Sunrise microplate spectrophotometer. The interaction between h-BN-OH-n in the cell culture medium and the LDH assay components was carried out in the absence of cells. The percentage of relative cell viability after 24-hour exposure was calculated using the following formula (2):

2 $$\begin{array}{c}{\rm{relative}}\,{\rm{cell}}\,{\rm{viability}}( \% )=100-({{\rm{A}}}_{490{\rm{nm}}}{\rm{of}}\,{\rm{treated}}\,{\rm{and}}\,{\rm{untreated}}\,{\rm{cells}}-{{\rm{A}}}_{490{\rm{nm}}}{\rm{of}}\\ {\rm{control}}/{{\rm{A}}}_{490{\rm{nm}}}{\rm{of}}\,{\rm{maximum}}\,{\rm{of}}\,{\rm{untreated}}\,{\rm{cells}}-{{\rm{A}}}_{490{\rm{nm}}}{\rm{of}}\,{\rm{control}})\times 100({\rm{A}}\,{\rm{is}}\,{\rm{absorbance}})\end{array}$$

#### Relative mitochondrial activity assay

The relative mitochondrial activity of the L929 cells (seeded at a density of 1 × 10^4^/well 24 hours before experimental exposure) was determined after a 24-hour incubation with h-BN-OH-n (in the range of 0.0–200.00 μg/ml) using a cell counting kit-8 (CCK-8, Sigma-Aldrich, St. Louis, MO, USA). The CCK-8 solution was added to each well of 96-well plates and incubated for 2 hours at 37 °C under standard culture conditions. The optical density (OD) was recorded at 450 nm (with the reference wavelength at 630 nm), according to the manufacturer’s instructions, using a Sunrise absorbance reader (Tecan, Männedorf, Switzerland). All experiments were conducted in triplicate. The effect of h-BN-OH-n on mitochondrial activity was calculated using the following formula (3):

3 $${\rm{relative}}\,{\rm{viability}}( \% )=({\rm{sample}}\,{{\rm{A}}}_{450-630{\rm{nm}}}/{\rm{positive}}\,{\rm{control}}\,{{\rm{A}}}_{450-630{\rm{nm}}})\times 100({\rm{A}}\,{\rm{is}}\,{\rm{absorbance}})$$

#### Neutral red uptake assay

The neutral red uptake assay (*in vitro* toxicology assay kit, Sigma-Aldrich, St. Louis, MO, USA) was performed 24 hours after the L929 cells were exposed to h-BN-OH-n. This assay is based on the ability of live cells to store the neutral red dye in acidic organelles (such as lysosomes) by active transport. Fresh DMEM containing 10% neutral red was added to the cultures and incubated at 37 °C in 5% CO_2_ and 95% relative humidity in an incubator for 3 hours. After incubation, the cells were washed twice with DPBS, and the solubilization solution was added to release the incorporated dye from the cellular organelles. The L929 cells were allowed to rest for 10 min at room temperature and then gently stirred, and the absorbance at 540 nm (the background absorbance at 690 nm) was determined using a Sunrise microplate. The effect of h-BN-OH on lysosomal activity was calculated using the following formula (4):

4 $${\rm{cell}}\,{\rm{relative}}\,{\rm{viability}}( \% )=({\rm{sample}}\,{{\rm{A}}}_{540{\rm{nm}}-690{\rm{nm}}}/{\rm{positive}}\,{\rm{control}}\,{{\rm{A}}}_{540{\rm{nm}}-690{\rm{nm}}})\times 100({\rm{A}}\,{\rm{is}}\,{\rm{absorbance}})$$

### Statistical analysis

For the statistical analysis of data, we used GraphPad Prism 5 software. Statistical analyses were performed using Student’s *t*-tests. All data were considered statistically significant at p-values <0.05.

## Data Availability

The data sets used during the current study are available from the corresponding author on reasonable request.
